# IL-2, IL-15 and IL-21 expand T cells for targeted adoptive therapy

**DOI:** 10.1186/2051-1426-3-S2-P31

**Published:** 2015-11-04

**Authors:** Zhenjiang Liu, Qingda Meng, Markus Maeurer, Elena Rangelova, Thomas Poiret, Rebecca Robertson, Aditya Ambati, Lalit Rane, Jiri Bartek, Caroline Verbeke, Oscar Persson, Matthias Löhr, Ralf Segersvärd, Ernest Dodoo

**Affiliations:** 1 Karolinska Institutet, Stockholm, Sweden

**Keywords:** TAA, T cells, IL-2, IL-15, IL-21, adoptive T cell therapy

## Background

Expansion of antigen-specific T cells, from peripheral blood specific for tumor-associated antigens (TAAs) is a prerequisite for the advanced cellular therapy. Such antigen-specific T cells should express a Th1-functional phenotype and are able to enter tumor-tissue. We identified a cytokine cocktail, comprised of IL-2, IL-15 and IL-21 that drives antigen-specific T cells from peripheral blood mononuclear cells to tumor-associated antigens.

## Materials and methods

20 blood samples (3 from health donors, 11 from glioma patients and 6 from pancreatic cancer patients) were collected, T cells were expanded using the cytokine cocktail IL-2/IL-15/IL-21 and TAAs, e.g. NY-ESO-1 or infectious antigens, e.g. CMV pp65 within 18-21 days. Intracellular Cytokine Staining (ICS) was used to detect antigen-specific immune responses by combining CD3, CD4 and CD8 markers with IL-2, TNF-α, IFN-γ production. Chemokine markers like CCR4, CCR6 or CXCR3 were used for phenotyping distinguishing Th1 and Th2 subtypes in CD4+ T cells, as well as in CD3+, CD4-CD8- (double-negative) T cell subsets. T cell memory phenotypes were defined by CCR7 and CD45RA staining. Tetramer (NY-ESO-1 or CMV specific) staining were used to show expansion of antigen specific T cells using a panel of mutant MHC class I tetramer molecules that allow to gauge for high, intermediate and low-affinity T cell populations imposed by interference of the MHC class I heavy chain with the CD8α binding site.

## Results

T cells from peripheral blood could be expanded (up to 10e10 cells) using IL-2, IL-15 and IL-21 and TAA-specific CD8, CD4 and CD3+, CD4-CD8-T cells could reliably be expanded defined by intracellular cytokine staining. In general, Th1 cells (CCR4-CXCR3+CCR6-) could be readily expanded along with (i.e. from 22.2% to 92.1%) along with increased expression of CXCR3 in both CD8+ and CD4+ T cells that enables increased access to tumor tissue. MHC class I-reactive T cells, directed against single T cell epitopes could be observed in up to 10% of CD8+, NY-ESO-1 specific T cells with a strong expansion of ‘high affinity’ T cells defined by i) mutant tetramers that interfere with CD8 engagement and ii) TAA-reactive T cells in the CD3+, CD4-CD8-double negative T cell subset.

## Conclusions

T cells from peripheral blood samples can be reliably and successfully expanded in IL-2, IL-15 and IL-21, they show high affinity TCR – MHC class I/peptide engagement, a Th1-cytokine production pattern and increased CXCR3 expressing allowing T cell access into tissues. A Phase I clinical trial to target TAAs in patients with glioblastoma or pancreatic cancer will soon start at Karolinska using IL-2/IL-15/IL-21 and TAA-expanded T cells.

